# Basic principles of sensorimotor adaptation to different distortions with different effectors and movement types: a review and synthesis of behavioral findings

**DOI:** 10.3389/fnhum.2013.00081

**Published:** 2013-03-14

**Authors:** Otmar Bock

**Affiliations:** Institute of Physiology and Anatomy, German Sport UniversityKöln, Germany

**Keywords:** motor learning, plasticity, context-dependence, transfer, multiple adaptation

## Abstract

This article reviews seemingly conflicting behavioral data about sensorimotor adaptation. Some earlier studies assert that one common mechanism exists for multiple distortions, and others that multiple mechanisms exist for one given distortion. Some but not others report that adaptation is direction-selective. Some submit that adaptation transfers across effectors, and others that a single effector can adapt to multiple distortions. A model is proposed to account for all these findings. It stipulates that adaptive mechanisms respond to multiple distortions, consist of directionally tuned special-purpose modules, can be switched in dependence on contextual cues, and are connected to practiced movement types with a higher weight than to unpracticed ones.

Human sensorimotor adaptation has been evaluated with a baffling number of experimental paradigms. Subjects were exposed to distortions of visual (Stratton, 1897), acoustic (Mikaelian, 1974) and proprioceptive inputs (Lackner and DiZio, 1994), to topographical (Kohler, 1955; Cunningham and Welch, 1994) and to dynamical distortions (Shadmehr and Mussa-Ivaldi, 1994; Bock, 2003), to distortions experienced while tracking (Cunningham and Welch, 1994), pointing (Mikaelian, 1974) or grasping with the hand (Gentilucci et al., 1995; Weigelt and Bock, 2007), while executing pursuit eye movements (Carl and Gellman, 1986), reflexive (McLaughlin, 1967) or volitional saccades (Deubel, 1995). Given this wealth of paradigms, it seems reasonable to question whether all authors dealt with the same phenomenon: is all adaptation achieved by one common mechanism, or rather by multiple mechanisms, each specific for a given paradigm?

This question has been addressed in behavioral studies by testing for the transfer of adaptation from one visual rotation to another, or from one lateral shift to another. This work invariably found that subjects started under the second distortion with the behavior they acquired under the first, and then gradually modified it until it became adequate for the second distortion; as a consequence, they performed better than novices when the second distortion was *larger* than the first, but worse than novices when the second distortion was *opposite* to the first (Lazar and van Laer, 1968; Wigmore et al., 2002; Bock et al., 2003). Thus transfer was compulsory, occurring even where it degraded performance. Other work found compulsory transfer even between distortions of a different type, i.e., between a visual rotation and a visual velocity-dependent lateral shift (Thomas and Bock, 2010), between a visual rotation and a force field (Bock and Thomas, 1999), and between a visual and an acoustic rotation (Kagerer and Contreras-Vidal, 2009). In those studies, performance benefits again emerged when both distortions were of equal sign, and costs when they were of opposite sign. Taken together, these findings suggest that adaptation to a wide range of distortions might be based on a common mechanism; this is illustrated in **Figure 1A**, where a universal adaptive mechanism receives sensory inputs *I*_j_ from different sensory modalities distorted in different ways, and sends motor outputs *O*_k_ to different effectors executing different types of movement.

**FIGURE 1:**
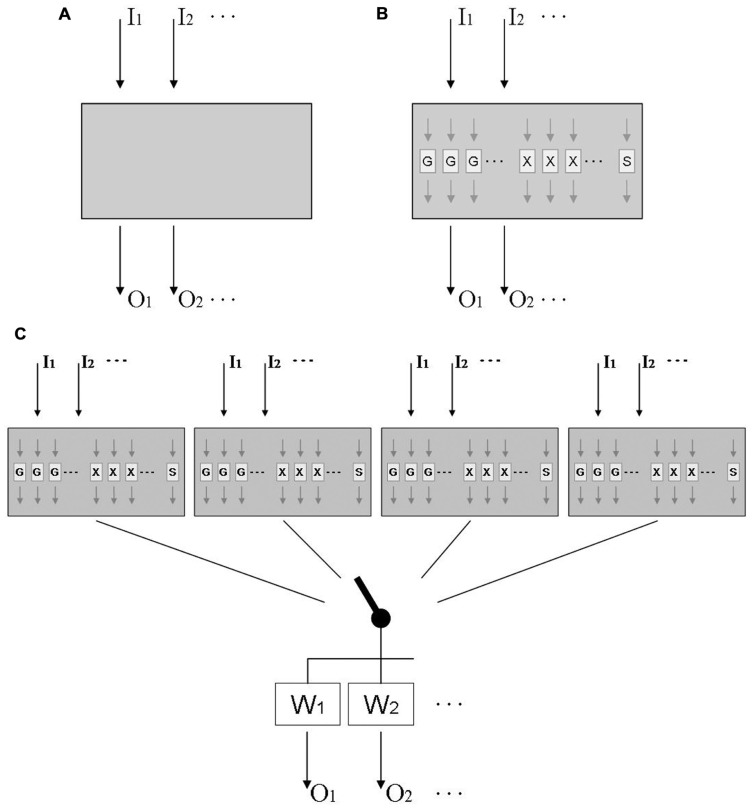
**Tentative models of sensorimotor adaptation**. **(A)** Model of an adaptive mechanism that receives inputs *I*_k_ from different sensory modalities distorted in different ways, and sends outputs *O*_k_ to different effectors executing different types of movement. **(B)** More elaborate model that includes functional modules *G*_i_ for gradual changes, *X*_i_ for axis inversions, and S for scaling; modules *G*_i_ and *X*_i_ are laid out in parallel, each being tuned to a limited range of target directions. **(C)** Final model that includes multiple mechanisms linked to the motor output by a context-dependent switch, and weighting factors that are higher for practiced than for unpracticed effectors and movement types.

Other findings have refined this view by indicating that the proposed universal mechanism can be subdivided into several functionally specialized modules. Thus, subjects exposed to different visual rotations perform less and less well as the magnitude of rotation increases toward 90°, but improve again as rotation continues to increase from 90° toward 180°; in fact, performance under a 180° rotation is not dramatically poorer than under no rotation (Cunningham, 1989; Abeele and Bock, 2001). Furthermore, subjects exposed to a rotation of more than 90° quickly change their response direction by 180° and then gradually change it “back” toward the required angle (Bock et al., 2003). These findings call for the existence of two functional modules, one that gradually changes spatial coordinates by up to 90°, and a second one that quickly changes them by 180°; the latter module possibly exploits the mathematical equivalence between a 180° rotation and an inversion of the horizontal and vertical axis.

Further work suggests that the presumed gradual-change modules are selective to only a limited range of movement directions around the practiced direction (Krakauer et al., 2000; Wang and Sainburg, 2005). This range can be estimated from published data as 45° (Tanaka et al., 2009) to 80° (Roby-Brami and Burnod, 1995), which fits well with the finding that adaptation shows only modest signs of interference when eight targets, located 45° apart, are associated with different rotational transformations (Werner and Bock, 2010). We posit that the axis-inversion modules are direction-selective as well, i.e., they operate only for movement directions similar to the trained ones; however, this issue has not been addressed experimentally yet. In contrast, adaptation to a new scaling factor seems not to be directionally tuned: adaptation of one movement direction transfers obligatorily to the full 360° range of possible directions (Bock, 1992; Krakauer et al., 2000). **Figure 1B** therefore depicts an adaptive mechanism that responds to multiple distortions with a number of special-purpose modules: several directionally tuned ones for gradual changes of direction (*G*_i_), several directionally tuned ones for axis inversions (*X*_j_), and a single one for scaling (S). This layout correctly predicts the obligatory transfer between distortions, the concurrence of quick and gradual changes under one given distortion, and the distinct adaptation characteristics with rotations and scalings.

The interplay of special-purpose modules such as those in **Figure 1B** can be readily illustrated with available data on the adaptation to mirror-reversed vision. This distortion initiates quick 180° changes of response directions for targets presented at the left and right, quick 180° changes followed by gradual 90° clockwise changes for targets along the right diagonal, quick 180° changes followed by gradual 90° counter-clockwise changes for targets along the left diagonal, and only a transient increase of response variability for targets at the top and bottom (Werner and Bock, 2010). This pattern of findings can be easily explained by the model in **Figure 1B**: targets at the left, right, and along either diagonal activate the corresponding directionally tuned axis-inversion modules, and targets along the diagonals additionally activate the corresponding gradual-change modules. Note that such an interpretation puts the minimum number of gradual-change modules to eight: the distortion activates four modules tuned to the diagonal directions, and has no effect on four modules tuned to the interleaved orthogonal directions. As noted above, this number of modules fits well with their reported tuning width of 40–80°, since 360/8 = 45. Similarly, the minimum number of axis-inversion modules seems to be 4: the distortion activates modules at the right and left, but not those at the top and bottom. For reasons of parsimony, one might therefore postulate eight gradual-change and four axis-inversion modules, but for reasons of symmetry, one might postulate eight modules of either type. Further research is needed to resolve this issue.

Adaptation to a given distortion does not transfer well to unpracticed movement types. A moderate transfer was observed between manual tracking and pointing (Abeele and Bock, 2003; Bock, 2005), grasping and pointing (Weigelt and Bock, 2010), as well as volitional saccades and pointing (Cotti et al., 2007), but no transfer was found between reactive and volitional saccades (Deubel, 1995), nor between reactive saccades and pointing (Cotti et al., 2007). Transfer between the two arms varied widely between studies and seems not to be obligatory, since both arms can concurrently adapt to opposite visual rotations with no sign of interference (Prablanc et al., 1975; Wang and Sainburg, 2003; Bock et al., 2005). Similarly, manual pointing and reactive saccades can concurrently adapt to two opposite distortions with only moderate interference (Grigorova et al., 2013). It even has been shown that one single arm, pointing at a single set of targets, can concurrently adapt to two opposite distortions if they are coded by contextual cues such as hemi-workspace (Ghahramani and Wolpert, 1997; Woolley et al., 2007), head position (Seidler et al., 2001), or screen color (Wada et al., 2003). In fact, subjects can adapt with no noticeable interference to as many as *four* distortions, each coded by a unique combination of arm and hemi-workspace (Thomas and Bock, 2012). Even when contextual cues are not available, subjects can use a “probing” movement to find out whether a previously established adaptive change should be preserved or rather abandoned (Wang and Sainburg, 2003). To account for these findings, **Figure 1C** shows four distinct multi-distortion mechanisms that can be alternately connected to the motor output via a context-dependent switch; the signal is then weighted, with the trained effector and movement type receiving the highest weight.

A model of sensorimotor adaptation, consisting of multiple mechanisms that are selectable by context, has been proposed before (Ghahramani and Wolpert, 1997; Wolpert and Kawato, 1998). The present article refines this model by adding multi-distortion sensitivity, special-purpose modules, directional tuning, and output weighting. The available database provides robust evidence for the existence of these key characteristics of adaptation, but future experimental findings may require an increase in the number of adaptive mechanisms and/or special-purpose modules. Additional research is also desirable to find out whether adaptive mechanisms are truly universal, i.e., respond to any conceivable type of distortion, and to determine the actual tuning widths of modules and weights of outputs. This would allow a quantitative rather than qualitative comparison of experimental data with model predictions.

The model in **Figure 1C** was designed to illustrate the known functional characteristics of adaptation; it was not meant to show the actual anatomical layout of the underlying neuronal circuitry. In fact, given the preponderance of parallel distributed processing in the brain, it is quite likely that the depicted modules and mechanisms are implemented within a highly interconnected neural network with only a limited topographical segregation. In a way, the model in **Figure 1C** could be interpreted as a specific version of schema theory, which posits that movements are executed by tailoring a generalized motor program to the needs of a specific movement (Schmidt, 1975).

As complex as it is, the model proposed in **Figure 1C** still disregards two crucial aspects of sensorimotor adaptation. One of them is the existence of multiple time scales. Gradual rotation proceeds with a time constant τ_1_ in the order of several movements, and a second one with a time constant τ_2_ in the order of several tens of movements (Snoddy, 1926; Smith et al., 2006); additional time scales in the order of days to months have been reported by classical accounts (Stratton, 1897; Kohler, 1955) and by recent spaceflight studies (Bock et al., 2010; Gaveau et al., 2011; Mulavara et al., 2012). Since the model in **Figure 1C** is mainly based on findings about long-term adaptation, it most likely represents the τ_2_ component. Little is known about the characteristics of the τ_1_ component, except that it acts in parallel rather than in series to τ_2_ (Lee and Schweighofer, 2009), requires working-memory resources (Anguera et al., 2010), is context-independent (Lee and Schweighofer, 2009) and exhibits its own distinctive directional tuning (Bock and Schmitz, 2011). It still is unknown whether axis inversion and scaling also proceeds along multiple time scales.

The second neglected aspect is the contribution of strategies. Exposure to a distortion initiates not only the adaptive recalibration of sensorimotor pathways, but also the use of workaround strategies such as cognitive reinterpretations of sensory signals, anticipations, associative stimulus–response learning, postural changes, and error-based corrections (Redding and Wallace, 1996; McNay and Willingham, 1998; Clower and Boussaoud, 2000). These strategies are thought to be situation-specific and short-lived, and thus to modify performance during exposure to a distortion, but not after removal of the distortion or after transfer to a new movement type. Evidence for the role of strategies is therefore largely based on the dissociated effects of higher-order mental functions on subjects’ performance *during* but not *after* exposure, e.g., the effects of aging (McNay and Willingham, 1998; Bock, 2005), emotional state (Bock, 2010), and explicit knowledge (Werner and Bock, 2007).

Summing up, **Figure 1C** presents a model for the slow component of adaptive recalibration that accounts for a wide range of seemingly contradictory behavioral phenomena: compulsory versus partial versus null transfer, common mechanism for multiple distortions versus multiple mechanisms for one distortion, presence versus absence of direction-selectivity, and eye–arm transfer versus multiple adaptation of a single arm. Additional experiments are needed to verify the model, determine its parameter values, and possibly add further functional details.

## Conflict of Interest Statement

The author declares that the research was conducted in the absence of any commercial or financial relationships that could be construed as a potential conflict of interest.

## References

[B1] Abeele S. Bock O. (2001). Sensorimotor adaptation to rotated visual input: different mechanisms for small versus large rotations. *Exp. Brain Res.* 140 407–410 11685393 10.1007/s002210100846

[B2] Abeele S. Bock O. (2003). Transfer of sensorimotor adaptation between different movement categories. *Exp. Brain Res.* 148 128–132 12478403 10.1007/s00221-002-1317-0

[B3] Anguera J. A. Reuter-Lorenz P. A. Willingham D. T. Seidler R. D. (2010). Contributions of spatial working memory to visuomotor learning. *J. Cogn. Neurosci.* 22 1917–1930 19803691 10.1162/jocn.2009.21351

[B4] Bock O. (1992). Adaptation of aimed arm movements to sensorimotor discordance: evidence for direction-independent gain control. *Behav. Brain Res.* 51 41–50 1482544 10.1016/s0166-4328(05)80310-9

[B5] Bock O. (2003). Sensorimotor adaptation to visual distortions with different kinematic coupling. *Exp. Brain Res.* 151 557–560 12856094 10.1007/s00221-003-1553-y

[B6] Bock O. (2005). Components of sensorimotor adaptation in young and elderly subjects. *Exp. Brain Res.* 160 259–263 15565436 10.1007/s00221-004-2133-5

[B7] Bock O. (2010). Sensorimotor adaptation is influenced by background music. *Exp. Brain Res.* 203 737–741 20480363 10.1007/s00221-010-2289-0

[B8] Bock O. Abeele S. Eversheim U. (2003). Human adaptation to rotated vision: interplay of a continuous and a discrete process. *Exp. Brain Res.* 152 528–532 14513189 10.1007/s00221-003-1643-x

[B9] Bock O. Bloomberg J. J. Weigelt C. (2010). Cognitive demand of human sensorimotor performance during an extended space mission: a dual-task study. *Aviat. Space Environ. Med.* 81 819–824 20824987 10.3357/asem.2608.2010

[B10] Bock O. Schmitz G. (2011). Adaptation to rotated visual feedback depends on the number and spread of target directions. *Exp. Brain Res.* 209 409–413 21279631 10.1007/s00221-011-2564-8

[B11] Bock O. Thomas M. (1999). “Sensorimotor adaptation to visual and mechanical perturbations is governed by a common mechanism.” FENS Meeting Wien 1999

[B12] Bock O. Worringham C. Thomas M. (2005). Concurrent adaptations of left and right arms to opposite visual distortions. *Exp. Brain Res.* 162 513–519 15754178 10.1007/s00221-005-2222-0

[B13] Carl J. R. Gellman R. S. (1986). “Adaptive responses in human smooth pursuit,” in *Adaptive Processes in Visual and Oculomotor Systems* eds Keller E. L. Zee D. S. (Oxford: Pergamon Press) 335–339

[B14] Clower D. M. Boussaoud D. (2000). Selective use of perceptual recalibration versus visuomotor skill acquisition. *J. Neurophysiol.* 84 2703–2708 11068013 10.1152/jn.2000.84.5.2703

[B15] Cotti J. Guillaume A. Alahyane N. Pélisson D. Vercher J.-L. (2007). Adaptation of voluntary saccades, but not of reactive saccades, transfers to hand pointing movements. *J. Neurophysiol.* 98 602–612 17553949 10.1152/jn.00293.2007

[B16] Cunningham H. A. (1989). Aiming error under transformed spatial mappings suggests a structure for visual-motor maps. *J. Exp. Psychol. Hum. Percept. Perform.* 15 493–506 2527958 10.1037//0096-1523.15.3.493

[B17] Cunningham H. A. Welch R. B. (1994). Multiple concurrent visual-motor mappings: implication for models of adaptation. *J. Exp. Psychol.* 20 987–999 10.1037//0096-1523.20.5.987 7964533

[B18] Deubel H. (1995). Separate adaptive mechanisms for the control of reactive and volitional saccadic eye movements. *Vis. Res.* 35 3529–3540 8560817 10.1016/0042-6989(95)00058-m

[B19] Gaveau J. Paizis C. Berret B. Pozzo T. Papaxanthis C. (2011). Sensorimotor adaptation of point-to-point arm movements after spaceflight: the role of internal representation of gravity force in trajectory planning. *J. Neurophysiol.* 106 620–629 21562193 10.1152/jn.00081.2011

[B20] Gentilucci M. Daprati E. Toni I. Chieffi S. Saetti M. C. (1995). Unconscious updating of grasp motor program. *Exp. Brain Res.* 105 291–303 7498382 10.1007/BF00240965

[B21] Ghahramani Z. Wolpert D. M. (1997). Modular decomposition in visuomotor learning. *Nature* 386 392–395 9121554 10.1038/386392a0

[B22] Grigorova V. Bock O. Ilieva M. Schmitz G. (2013). Directional adaptation of reactive saccades and of hand pointing movements is not independent. *J. Motor Behav.* 45 101–106 10.1080/00222895.2012.750590 23441689

[B23] Kagerer F. A. Contreras-Vidal J. L. (2009). Adaptation of sound localization induced by rotated visual feedback in reaching movements. *Exp. Brain Res.* 193 315–321 19048242 10.1007/s00221-008-1630-3 PMC3203351

[B24] Kohler I. (1955). Experiments with prolonged optical distortions. *Acta Psychol.* 11 176–178

[B25] Krakauer J. W. Pine Z. M. Ghilardi M.-F. Ghez C. (2000). Learning of visuomotor transformations for vectorial planning of reaching trajectories. *J. Neurosci.* 20 8916–8924 11102502 10.1523/JNEUROSCI.20-23-08916.2000 PMC6773094

[B26] Lackner J. R. DiZio P. (1994). Rapid adaptation to coriolis force perturbations of arm trajectory. *J. Neurophysiol.* 72 299–313 7965013 10.1152/jn.1994.72.1.299

[B27] Lazar G van Laer J. (1968). Adaptation to displaced vision after experience with lesser displacements. *Percept. Motor Skills* 26 579–582 5654890 10.2466/pms.1968.26.2.579

[B28] Lee J. Y. Schweighofer N. (2009). Dual adaptation supports a parallel architecture of motor memory. *J. Neurosci.* 29 10396–10404 19692614 10.1523/JNEUROSCI.1294-09.2009 PMC2789989

[B29] McLaughlin S. C. (1967). Parametric adjustment in saccadic eye movements. *Percept. Psychophys.* 2 359–362

[B30] McNay E. C. Willingham D. B. (1998). Deficit in learning of a motor skill requiring strategy, but not of perceptual motor recalibration, with aging. *Learn. Mem.* 4 411–420 10701880 10.1101/lm.4.5.411

[B31] Mikaelian H. H. (1974). Adaptation to displaced hearing: a nonproprioceptive change. *J. Exp. Psychol.* 103 326–330

[B32] Mulavara A. P. Ruttley T. Cohen H. S. Peters B. T. Miller C. Brady R. (2012). Vestibular-somatosensory convergence in head movement control during locomotion after long-duration space flight. *J. Vestib. Res.* 22 153–166 23000615 10.3233/VES-2011-0435

[B33] Prablanc C. Tzavaras A. Jeannerod M. (1975). Adaptation of the two arms to opposite prism displacements. *Q. J. Exp. Psychol.* 27 667–671

[B34] Redding G. M. Wallace B. (1996). Adaptive spatial alignment and strategic perceptual-motor control. *J. Exp. Psychol. Hum. Percept. Perform.* 22 379–394 8934851 10.1037//0096-1523.22.2.379

[B35] Roby-Brami A. Burnod Y. (1995). Learning a new visuomotor transformation: error correction and generalization. *Cogn. Brain Res.* 2 229–242 10.1016/0926-6410(95)90014-4 8580736

[B36] Schmidt R. A. (1975). A schema theory of motor learning. *Psychol. Rev.* 82 225–260

[B37] Seidler R. D. Bloomberg J. J. Stelmach G. E. (2001). Context-dependent arm pointing adaptation. *Behav. Brain Res.* 119 155–166 11165331 10.1016/s0166-4328(00)00347-8

[B38] Shadmehr R. Mussa-Ivaldi F. A. (1994). Adaptive representation of dynamics during learning of a motor task. *J. Neurosci.* 14 3208–3224 8182467 10.1523/JNEUROSCI.14-05-03208.1994 PMC6577492

[B39] Smith M. A. Ghazizadeh A. Shadmehr R. (2006). Interacting adaptive processes with different timescales underlie short-term motor learning. *PLoS Biol.* 4: e179 10.1371/journal.pbio.0040179 PMC1463025 16700627

[B40] Snoddy G. S. (1926). Learning and stability. *J. Appl. Psychol.* 10 1–36

[B41] Stratton G. M. (1897). Vision without inversion of the retinal image. *Psychol. Rev.* 4 341–481

[B42] Tanaka H. Sejnowski T. J. Krakauer J. W. (2009). Adaptation to visuomotor rotation through interaction between posterior parietal and motor cortical areas. *J. Neurophysiol.* 102 2921–2932 19741098 10.1152/jn.90834.2008 PMC2777823

[B43] Thomas M. Bock O. (2010). Is sensorimotor adaptation to position- and velocity-dependent visual distortions based on distinct adaptive processes? *Hum. Mov. Sci.* 29 179–186 20338653 10.1016/j.humov.2010.02.002

[B44] Thomas M. Bock O. (2012). Concurrent adaptation to four different visual rotations. *Exp. Brain Res.* 221 85–91 22777101 10.1007/s00221-012-3150-4 PMC3401298

[B45] Wada Y. Kawabata Y. Kotosaka S. Yamamoto K. Kitazawa S. Kawato M. (2003). Acquisition and contextual switching of multiple internal models for different viscous force fields. *Neurosci. Res.* 46 319–331 12804793 10.1016/s0168-0102(03)00094-4

[B46] Wang J. Sainburg R. L. (2003). Mechanisms underlying interlimb transfer of visuomotor rotations. *Exp. Brain Res.* 149 520–526 12677333 10.1007/s00221-003-1392-x PMC3697093

[B47] Wang J. Sainburg R. L. (2005). Adaptation to visuomotor rotations remaps movement vectors, not final positions. *J. Neurosci.* 25 4024–4030 15843604 10.1523/JNEUROSCI.5000-04.2005 PMC6724955

[B48] Weigelt C. Bock O. (2007). Adaptation of grasping responses to distorted object size and orientation. *Exp. Brain Res.* 181 139–146 17333005 10.1007/s00221-007-0911-6

[B49] Weigelt C. Bock O. (2010). Adaptation of the precision grip orientation to a visual–haptic mismatch. *Exp. Brain Res.* 201 621–630 20012539 10.1007/s00221-009-2076-y

[B50] Werner S. Bock O. (2007). Effects of variable practice and declarative knowledge on sensorimotor adaptation to rotated visual feedback. *Exp. Brain Res.* 178 554–559 17361424 10.1007/s00221-007-0925-0

[B51] Werner S. Bock O. (2010). Mechanisms for visuomotor adaptation to left–right reversed vision. *Hum. Mov. Sci.* 29 172–178 20304517 10.1016/j.humov.2010.02.004

[B52] Wigmore V. Tong C. Flanagan J. R. (2002). Visuomotor rotations of varying size and direction compete for single internal model in working memory. *J. Exp. Psychol. Hum. Percept. Perform.* 28 447–457 11999865 10.1037//0096-1523.28.2.447

[B53] Wolpert D. M. Kawato M. (1998). Multiple paired forward and inverse models for motor control. *Neural Netw.* 11 1317–1329 12662752 10.1016/s0893-6080(98)00066-5

[B54] Woolley D. G. Tresilian J. R. Carson R. G. Rick S. (2007). Dual adaptation to two opposing visuomotor rotations when each is associated with different regions of workspace. *Exp. Brain Res.* 179 155–165 17119942 10.1007/s00221-006-0778-y

